# Clinical Use and Effectiveness of Lipid Lowering Therapies in Diabetes Mellitus—An Observational Study from the Swedish National Diabetes Register

**DOI:** 10.1371/journal.pone.0018744

**Published:** 2011-04-29

**Authors:** Björn Eliasson, Ann-Marie Svensson, Mervete Miftaraj, Junmei Miao Jonasson, Katarina Eeg-Olofsson, Karolina Andersson Sundell, Soffia Gudbjörnsdóttir

**Affiliations:** 1 Department of Medicine, University of Gothenburg, Sahlgrenska University Hospital, Göteborg, Sweden; 2 National Diabetes Register (NDR), Göteborg, Sweden; 3 Department of Oncology, Institute of Clinical Sciences, University of Göteborg, Göteborg, Sweden; 4 Nordic School of Public Health, Göteborg, Sweden; Lerner Research Institute, Cleveland Clinic, United States of America

## Abstract

**Objectives:**

To describe the use and evaluate the effectiveness of different lipid lowering therapies in unselected patients with type 1 and type 2 diabetes in clinical practice.

**Design:**

Observational population-based study using the personal identification number to link information from the National Diabetes Register, the Prescribed Drug Register and the Patient register in Sweden. All patients in the NDR aged 18–75 years with diabetes more than one year were eligible, but only patients starting any lipid lowering treatment with at least three prescriptions 1 July 2006–30 June 2007 were included (n = 37182). The mean blood lipid levels in 2008 and reductions in LDL cholesterol were examined.

**Results:**

Blood lipid levels were similar in patients treated with simvastatin, atorvastatin and rosuvastatin, showing similar lipid lowering effect as currently used. Users of pravastatin, fluvastatin, ezetimib and fibrate more seldom reach treatment goals. Moderate daily doses of the statins were used, with 76% of simvastatin users taking 20 mg or less, 48% of atorvastatin users taking 10 mg, 55% of pravastatin users taking 20 mg, and 76% of rosuvastatin users taking 5 or 10 mg.

**Conclusions:**

This observational study shows that the LDL-C levels in patients taking simvastatin, atorvastatin or rosuvastatin are very similar as currently used, as well as their LDL-C lowering abilities. There is potential to intensify lipid lowering treatment to reduce the remaining high residual risk and achieve better fulfilment of treatment goals, since the commonly used doses are only low to moderate.

## Introduction

Recent randomized clinical trials and a major meta-analysis have emphasized the importance of LDL-cholesterol (LDL-C) lowering for cardiovascular risk reduction in diabetes mellitus [1]–[3]. Therefore the current treatment guidelines advocate aggressive multifactorial risk factor intervention in patients with diabetes [4], [5]. The European guidelines promote lifestyle changes and lipid lowering therapy in order to reach a lower LDL-C value than 2.5 mmol/L, or 1.8 mmol/L or lower if overt cardiovascular disease (CVD) is present [5]. The pharmacological treatment should be based on HMG CoA reductase inhibitors, also known as statins, but other options are to be considered if the treatment goals are not reached.

The LDL-C lowering effects of the different statins in clinical trials have recently been reviewed [6]. A small or moderate dose of statins could decrease LDL-C by 20–40%, with small differences between the different agents. These conclusions are in agreement with the CURVES and the STELLAR studies, in which atorvastatin and rosuvastatin, respectively, showed similar effects as other statin [7], [8]. At higher doses, however, atorvastatin and rosuvastatin are the only agents that can lower LDL-C more than 40% [6]. There have not been any randomized clinical trials or observational epidemiological studies with head to head comparisons of the cholesterol lowering effect by different statins in patients with diabetes.

The aim of this observational study linking data from the Swedish National Diabetes Register (NDR), a quality register with nation-wide coverage, with two other national population-based registers, was to describe the use and evaluate the LDL-C lowering effects of different lipid lowering therapies in 37 182 unselected patients with type 1 and type 2 diabetes in clinical practice.

## Methods

This is a population-based study using the personal identification number to link information from three national registers. NDR was initiated in 1996 as a tool for quality improvement in diabetes care, and has been described previously [9], [10]. Physicians and nurses in hospital outpatient clinics and primary health care clinics report to the NDR at least once every year, either online or by direct transfer of data from medical records databases. The Swedish Prescribed Drug Register contains information about dispensed prescribed drugs in the entire Swedish population of 9.4 million inhabitants [11]. The Swedish Patient Register contains information on dates of hospital admission and discharge, codes for all surgical procedures and discharge diagnoses [12], [13]. The Regional Ethical Review Board of the University of Gothenburg approved the study, and all included patients have agreed to be reported.

All patients aged 18–75 years in the NDR with diabetes for more than one year were eligible, but only patients who had not purchased any lipid lowering medicine 1 July 2005–30 June 2006 and thereafter filled at least three prescriptions 1 July 2006–30 June 2007 were included in the study (n = 37 182]. These criteria were chosen based on the Swedish Pharmaceutical Benefits Scheme where the patients normally fill a prescription for 90 days of supply, and can refill again when two thirds of the theoretical consumption time has passed. In some cases the first filled prescription encompasses only a small start package for 30 days of supply. Thus, those included in the study would have purchased lipid lowering drugs corresponding to seven months of use or more. Clinical characteristics including mean blood lipid values on treatment (2008) were studied in this group. We also performed a subgroup analysis of patients who also had a known LDL-C value between 1 July 2005 and 30 June 2006), i.e., before the initiation of lipid lowering therapy (n = 10 456).

The clinical characteristics analysed at baseline were age, sex, diabetes duration, BMI, smoking, blood pressure, HbA1c, total cholesterol (TC), HDL-C, and serum triglycerides. The patients were screened using local methods, but guidelines were available to ensure the use of similar methodology. A smoker was defined as a patient smoking one or more cigarettes per day, or a pipe daily, or who had stopped smoking within the past three months. Renal disease was defined as a history of acute, chronic, and any or unspecified renal insufficiency.

Laboratory analyses, including TC and HDL-C levels, were carried out at local laboratories. HbA1c analyses are quality assured in Sweden by regular calibration with Mono-S, a HPLC method. In this study, all HbA1c values were converted to the DCCT (Diabetes Control and Complications Trial) standard levels: HbA1c(DCCT) = 0.923×HbA1c(Mono-S)+1.345; R^2^ = 0.998 [14]. LDL-C was calculated using Friedewald's formula [15] if serum TG levels were lower than 4.0 mmol/L [16].

History of CVD recorded at hospital discharge was retrieved from the Swedish Patient Register. CVD was defined as diagnosis of myocardial infarction, angina pectoris, intracerebral haemorrhage, cerebral infarction or unspecified stroke before the survey, but peripheral vascular disease was not included.

### Statistical methods

General linear modelling was used to compare clinical characteristics and reductions in LDL-C. The relative risks of reaching LDL-C≥2.5 mmol/L were estimated by using generalized linear modelling and simvastatin as the reference. When adjusting for potential confounding factors, we categorised the numeric variables: age (<30 years, 30–39 years, 40–49 years, 50–59 years, ≥60 years), diabetes duration (<10 years, ≥10 years), LDL-C level before taking statin (<2.5 mmol/L, ≥2.5 mmol/L). Median doses of the lipid lowering agents were used as cut-offs in these calculations (high dosages: simvastatin ≥20 mg, pravastatin ≥40 mg, fluvastatin ≥40 mg, atorvastatin ≥20 mg, rosuvastatin ≥10 mg as high dosage, fibrates ≥0.5 mg; all used 10 mg ezetimib). In order to avoid a substantial reduction of the number of subjects, we accepted ‘missing value’ of LDL-C as a single category in our main analyses. All statistical analyses were performed by use of SAS statistical software version 9.2 (SAS Institute, Cary, NC, USA).

## Results

Table 1 gives the clinical characteristics of the patients on any lipid lowering treatment in 2008. Of all patients around 75% used simvastatin, 14% used atorvastatin, 4% used pravastatin, 3% used a statin plus ezetimib combination and 2% used a statin plus fibrate combination. Fluvastatin, rosuvastatin and ezetemib were used by 1% or less of the patients, respectively. The mean age was around 62 years with almost 15 years of mean diabetes duration. The proportion of men in the cohort was around 60%, circa 10% had type 1 diabetes and 13% were smokers. Mean BMI was almost 30 kg/m^2^, mean blood pressure was 135/75 mm Hg and HbA1c 6.4%. There were statistically significant differences between mean values and proportions of all risk factors (except diastolic blood pressure) in the different treatment groups and also between the users of the different statins (except systolic and diastolic blood pressure). A history of CVD was most common in patients on pravastatin, fluvastatin or rosuvastatin. In patients on simvastatin, a fibrate or combination therapy, a history of renal disease was less common.

**Table 1:** Clinical characteristics of the patients on lipid lowering treatment 2008.

Variable		Simvastatin	Pravastatin	Fluvastatin	Atorvastatin	Rosuva-statin	Ezetimib	Fibrate	Statin +fibrate	Statin +ezetimib	P-values: overall statins
Number of patients	N	28025	940	159	5098	355	208	536	754	1107	
Age	Mean±SD	62.8±8.6	64.7±7.4	64.3±7.9	62.6±8.2	60.8±8.7	62.9±8.2	62.2±8.5	62.1±8.2	61.7±8.6	<0.0001 <0.0001
Duration	N	28025	940	159	5098	355	208	536	754	1107	
	Mean±SD	12.8±11.5	14.2±12.0	15.5±13.7	14.5±12.1	12.5±11.4	15.5±13.2	12.8±8.8	11.9±8.6	13.2±12.1	<0.0001 <0.0001
Men	N	16444	519	102	3078	194	104	336	513	645	
	%	58.7	55.2	64.2	60.4	54.6	50.0	62.7	68.0	58.3	<0.0001 0.0051
Type 1 diabetes	N	2536	73	24	522	33	26	17	15	115	
	%	9.0	7.8	15.1	10.2	9.3	12.5	3.2	2.0	10.4	<0.0001 0.0027
Type 2 diabetes	N	23594	793	126	4148	291	167	465	671	897	
	%	84.2	84.4	79.2	81.4	82.0	80.3	86.8	89.0	81.0	<0.0001 <0.0001
Systolic blood pressure	N	27543	929	156	5011	348	204	530	736	1089	
	Mean±SD	136±16	137±16	137±17	136±16	135±16	136±16	138±17	136±17	135±16	0.0103 0.106
Diastolic blood pressure	N	27543	929	156	5011	348	204	530	736	1089	
	Mean±SD	75±9	75±9	75±10	75±9	75±10	76±9	77±10	75±9	75±9	0.1531 0.968
BMI	N	26569	883	148	4814	335	198	502	714	1047	
	Mean±SD	29.6±5.1	29.6±5.0	29.3±4.8	29.9±5.1	30.0±5.0	29.4±4.9	30.2±5.0	30.8±4.8	30.1±4.9	<0.0001 0.0002
Smokers	N	3659	121	13	676	44	19	68	127	152	
	%	13.0	12.9	8.2	13.3	12.4	9.1	12.7	16.8	13.7	0.0348 0.0506
HbA1c	N	27836	935	158	5072	354	208	531	748	1099	
		6.4±1.2	6.3±1.1	6.5±1.3	6.5±1.3	6.5±1.4	6.3±1.2	6.4±1.3	6.5±1.3	6.6±1.3	<0.0001 <0.0001
CVD	N	5567	258	44	1153	97	46	86	143	195	
	%	19.9	27.4	27.7	22.6	27.3	22.1	16.0	19.0	17.6	<0.0001 <0.0001
Renal disease	N	2472	107	39	641	48	29	37	63	99	
	%	8.8	11.4	24.5	12.6	13.5	13.9	6.9	8.4	8.9	<0.0001 <0.0001

CVD, history of cardiovascular disease; Renal disease, history of renal disease. SD, standard deviation.

The numbers of patients and the proportion of patients reaching LDL-C<2.5 mmol/L on the different doses of the statins are given in Table 2. In patients with LDL-C<2.5 mmol/L the distribution of doses were the same as in the overall cohort. Only ezetimib 10 mg was used. In patients on fibrates, a daily dose of 600 mg was the most common dose, used in 44% of these patients. In statin plus ezetimib or fibrate combination therapy, simvastatin was used in 64%, atorvastatin in 26%, pravastatin in 5% and rosuvastatin in 4%.

**Table 2:** Distribution of mean doses of the statins in patients on statins.

Substance	Number (N) and proportion (%)	Dose					
		5 mg	10 mg	20 mg	40 mg	80 mg	Total N
Simvastatin	N/%	n.a.	5457 (19,5%)	15874 (56,6%)	6592 (23,5%)	102 (0,4%)	28025
	% with LDL-C<2.5 mmol/L	n.a.	3292 (18.4%)	10220 (57.2%)	4319 (24.1%)	50 (0.3%)	17881
Pravastatin	N/%	n.a.	n.a.	515 (54,8%)	425 (45,2%)	n.a.	940
	% with LDL-C<2.5 mmol/L	n.a.	n.a.	218 (51.4%)	206 (48.6%)	n.a.	424
Fluvastatin	N/%	n.a.	n.a.	60 (37,7%)	47 (29,6%)	52 (32,7%)	159
	% with LDL-C<2.5 mmol/L	n.a.	n.a.	16 (26.2%)	16 (26.2%)	29 (47.6%)	61
Atorvastatin	N/%	n.a.	2466 (48,4%)	1713 (33,6%)	415 (8,1%)	504 (9,9%)	5098
	% with LDL-C<2.5 mmol/L	n.a.	1606 (48.9%)	1103 (33.6%)	252 (7.7%)	319 (9.8%)	3280
Rosuvastatin	N/%	17 (4,8%)	254 (71,5%)	75 (21,1%)	9 (2,5%)	n.a.	355
	% with LDL-C<2.5 mmol/L	13 (5.5%)	174 (73.1%)	47 (19.7%)	4 (1.7%)	n.a	238

N.a., not applicable.

**Figure 1:**
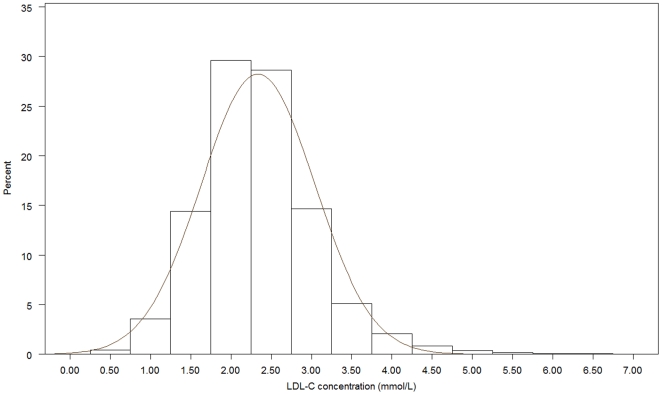
Histogram for the LDL-C values in patients on simvastatin.

**Figure 2:**
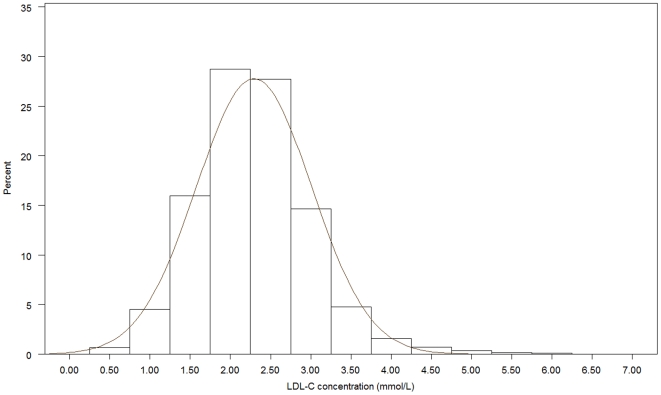
Histogram for the LDL-C values in patients on atorvastatin.

**Figure 3:**
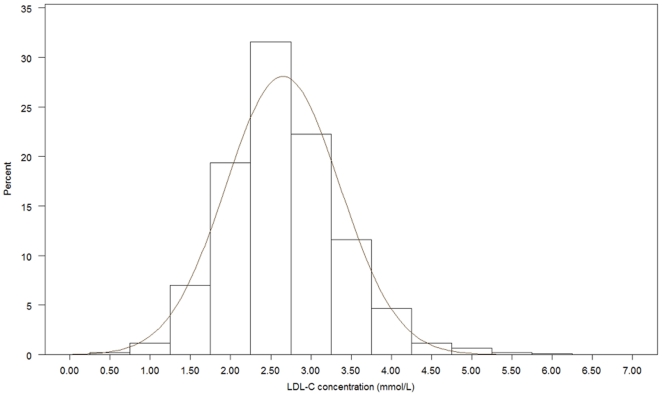
Histogram for the LDL-C values in patients on pravastatin.

**Figure 4:**
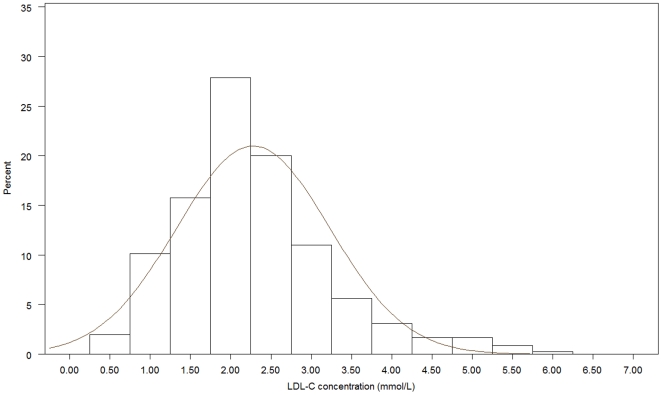
Histogram for the LDL-C values in patients on rosuvastatin.

**Figure 5:**
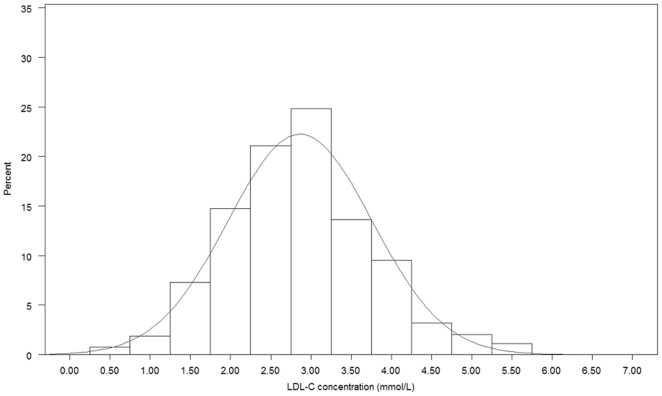
Histogram for the LDL-C values in patients on a fibrate.

**Figure 6:**
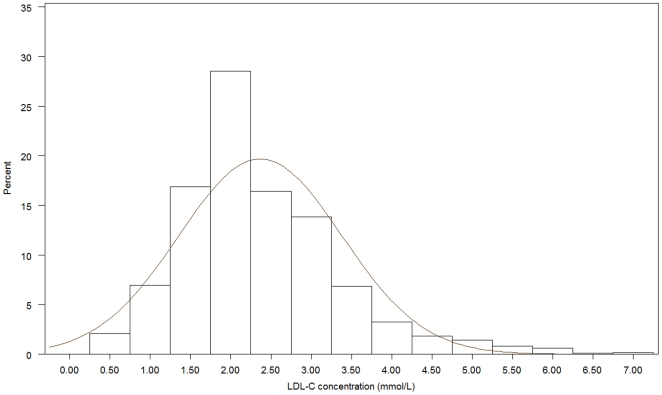
Histogram for the LDL-C values in patients on a statin and ezetimib combination.

In Table 3 blood lipid values are given and the proportion of patients achieving the current treatment goals. Figures 1, 2, 3, 4, 5, 6 presents the distribution of LDL-C values in patients on different lipid lowering treatments. TC, LDL-C were lower and the proportion of patients reaching the different treatment goals highest in patients on simvastatin, atorvastatin, rosuvastatin or a statin in combination with ezetimib or a fibrate. Consequently, the proportion of patients reaching LDL-C<2.5 mmol/L and ≤1.8 mmol/L (patients with a history of CVD) were highest (57.4%–67.0% and 20.3%–28.9%, respectively) in these five treatment groups. The group of patients on combination therapy or on fibrates only exhibited the highest TG levels and lowest HDL-C levels. The small group of patients on ezetimib only had the highest TC and LDL-C. The proportion of patients not reaching treatment goals of HDL-C were more than 60% in men and around 80% in women, while TG targets were not reached in 40–50% of the patients on the most frequently used statins.

**Table 3:** Blood lipid values of the patients on lipid lowering treatment 2008.

Variable		Simvastatin	Prava-statin	Fluva-statin	Atorva-statin	Rosuva-statin	Ezetimib	Fibrate	Statin + fibrate	Statin + ezetimib
TC	N	27887	933	158	5065	351	207	534	751	1097
	Mean±SD	4.4±0.8	4.7±0.8	4.8±0.9	4.4±0.8	4.4±1.1	5.3±1.0	4.9±1.0	4.5±0.9	4.5±1.2
TC<4.5	N	15758	367	59	2938	201	44	185	378	589
	%	56.5	39.3	37.3	58.0	57.3	21.2	34.6	50.3	53.7
LDL-C	N	28025	940	159	5098	355	208	536	754	1107
	Mean±SD	2.3±0.7	2.7±0.7	2.7±0.7	2.3±0.7	2.3±1.0	3.1±0.8	2.9±0.9	2.4±0.9	2.4±1.0
LDL-C<2.5	N	17881	424	61	3280	238	58	182	433	698
	%	63.8	45.1	38.4	64.3	67.0	27.9	34.0	57.4	63.0
LDL-C≤1.8 with history of CVD	N	1526	30	4	310	33	2	7	39	67
	%	25.9	32.2	25.0	25.7	28.9	25.0	12.1	21.08	20.3
HDL-C	N	27769	927	159	5052	347	206	533	748	1090
	Mean±SD	1.3±0.4	1.3±0.4	1.3±0.4	1.3±0.4	1.3±0.4	1.3±0.4	1.1±0.4	1.1±0.3	1.2±0.4
HDL-C>1.0 (men)	N	10752	326	56	1819	117	65	136	196	381
	%	38.7	35.2	35.2	36.0	33.7	31.6	25.5	26.2	35.0
HDL-C>1.3 (women)	N	5849	194	28	898	66	54	61	59	205
	%	21.1	20.9	17.6	17.8	19.0	26.2	11.4	7.9	18.8
TG	N	27661	924	159	5036	346	207	533	749	1090
	Mean±SD	1.7±0.9	1.7±0.8	1.8±0.9	1.8±0.9	1.9±1.0	1.8±1.0	2.1±1.2	2.4±1.3	2.1±1.2
TG<1.7	N	16309	493	82	2624	171	103	241	229	484
	%	59.0	53.4	51.6	52.1	49.4	49.8	45.2	30.6	44.4

SD, standard deviation; TC, total cholesterol; LDL-C, LDL cholesterol; HDL-C, HDL cholesterol; TG, triglycerides.

Patients with type 1 diabetes were generally treated with simvastatin or atorvastatin (75% and 16%, respectively). The numbers of patients on the other lipid lowering treatments were very small (Table S1). In the simvastatin and atorvastatin treatment groups blood lipid levels were very similar, and as expected, with higher mean HDL-C and lower TG levels, while LDL-C (2.4±0.7 mmol/L) was numerically almost the same, as the overall cohort (2.3±0.7 mmol/L). In the patients with type 1 diabetes on simvastatin or atorvastatin, a history of CVD was less common but a history of renal disease clearly more prevalent (22.8% in simvastatin-treated patients and 32.4% in patients taking atorvastatin) than the overall cohort, which mainly consisted of patients with type 2 diabetes (79–89% in the different treatment groups).

The mean effects on LDL-C levels after starting a lipid lowering treatment in a subgroup of patients with an LDL-value both before and during treatment are given in Table 4. The clinical characteristics of these patients did not differ markedly from the data presented in Table 1 and 3 (data not shown). The most pronounced effects were seen in patients starting on simvastatin, rosuvastatin, ezetimib or statin plus ezetimib combination. Compared with the LDL-C levels before treatment, all changes were statistically significant except for pravastatin and fluvastatin.

**Table 4:** LDL cholesterol values of patients on lipid lowering treatments before and on treatment in 2008.

Variable		Simvastatin	Pravastatin	Fluvastatin	Atorva-statin	Rosuva-statin	Ezetimib	Fibrate	Statin + fibrate	Statin + ezetimib
Number of patients	N	7975	260	33	1398	83	64	138	215	290
LDL-C before lipid lowering treatment (mmol/L)	Mean±SD	2.6±0.9	2.7±0.7	2.7±0.8	2.4±0.8	2.6±1.0	3.3±0.9	3.0±0.9	2.5±0.9	2.8±1.1
LDL-C on lipid lowering treatment (mmol/L)	Mean±SD	2.3±0.7	2.7±0.7	2.7±0.7	2.3±0.7	2.3±1.0	3.1±0.8	2.9±0.9	2.4±0.9	2.4±1.0
Change (mmol/L)	Mean (95% CI)	0.24 (0.22–0.26)	0.05 (−0.02–0.13)	−0.002 (−0.18–0.17)	0.064 (0.02–0.10)	0.34 (0.12–0.56)	0.34 (0.10–0.59)	0.14 (0.01–0.27)	0.18 (0.07–0.29)	0.37 (0.25–0.49)
Change (%)	Mean	9.2	1.9	0.1	2.7	13.1	10.3	4.7	7.2	13.2
P-value		<0.0001	0.1654	0.9754	<0.0031	<0.0024	0.0063	0.0270	0.0009	<0.0001

SD, standard deviation; TC, total cholesterol; LDL-C, LDL cholesterol.

Table 5 gives the relative risks (and 95% confidence interval) of achieving a LDL-C level ≥2.5 mmol/L in patients taking other lipid lowering agents than simvastatin with those using simvastatin as reference category. Without adjustment for covariates, dose and LDL-C levels before the lipid lowering treatment, only atorvastatin and rosuvastatin showed no difference in relative risk. The relative risks were significantly higher than 1 in all other treatment groups. An identical pattern was seen also after adjustment for the covariates separately or all simultaneously, including doses of the lipid lowering treatment and LDL-C before the treatment.

**Table 5:** Relative risks and 95% confidence interval of lipid level ≥2.5 mmol/L in patients taking other lipid lowering agents than simvastatin compared to taking simvastatin.

	Simvastatin	Pravastatin	Fluvastatin	Atorvastatin	Rosuvastatin	Ezetimib	Fibrates
Model		RR (95% CI)					
Not adjusted	Referent	1.52 (1.43–1.61)	1.70 (1.50–1.93)	0.99 (0.95–1.03)	0.91 (0.78–1.06)	1.99 (1.83–2.17)	1.82 (1.71–1.94)
Adjusted for:							
Age	Referent	1.54 (1.45–1.63)	1.69 (1.50–1.91)	0.98 (0.94–1.02)	0.90 (0.77–1.04)	1.96 (1.80–2.13)	1.79 (1.69–1.91)
Sex		1.50 (1.42–1.60)	1.71 (1.52–1.94)	0.99 (0.95–1.03)	0.91 (0.78–1.05)	1.97 (1.81–2.15)	1.83 (1.72–1.95)
Diabetes duration		1.52 (1.43–1.62)	1.71 (1.51–1.93)	0.99 (0.95–1.03)	0.90 (0.78–1.05)	2.02 (1.85–2.19)	1.84 (1.73–1.96)
Smoking		1.52 (1.43–1.61)	1.71 (1.51–1.93)	0.98 (0.95–1.02)	0.91 (0.78–1.06)	2.00 (1.83–2.18)	1.82 (1.71–1.94)
Dose		1.48 (1.39–1.57)	1.69 (1.49–1.91)	0.96 (0.92–1.00)	0.92 (0.79–1.07)	-	1.77 (1.66–1.89)
LDL-C levels before treatment		1.49 (1.41–1.57)	1.68 (1.50–1.88)	1.01 (0.97–1.05)	0.90 (0.78–1.04)	1.73 (1.61–1.86)	1.68 (1.59–1.78)
CVD		1.53 (1.44–1.63)	1.72 (1.52–1.94)	0.99 (0.95–1.03)	0.92 (0.79–1.07)	2.00 (1.84–2.18)	1.79 (1.69–1.91)
Renal disease		1.52 (1.43–1.61)	1.75 (1.54–1.97)	0.99 (0.95–1.03)	0.91 (0.78–1.06)	2.01 (1.84–2.18)	1.81 (1.70–1.93)
Several variables*		1.55 (1.47–1.65)	1.75 (1.55–1.97)	1.00 (0.96–1.04)	0.90 (0.78–1.05)	2.01 (1.85–2.18)	1.78 (1.67–1.89)
Several variables#		1.52 (1.43–1.61)	1.74 (1.54–1.95)	0.98 (0.94–1.02)	0.91 (0.78–1.06)	-	1.73 (1.62–1.84)

CVD, history of cardiovascular disease; Renal disease, history of renal disease. SD, standard deviation; RR, Relative risk; CI, Confidence intervals.

*Adjusted for age, duration of diabetes, smoking, CVD, renal diseases.

# Adjusted for age, duration of diabetes, smoking, LDL-level before the treatment, CVD, renal diseases.

## Discussion

This observational study examining clinical use and the effects on LDL-C levels of lipid lowering therapies shows that blood lipid levels are very similar in patients treated with simvastatin, atorvastatin and rosuvastatin in clinical practice in Sweden. These three agents have also shown similar LDL-C lowering effects as currently used. A combination with a statin and ezetimib or a fibrate also shows similar effects, while users of pravastatin, fluvastatin, ezetimib and fibrate more seldom reach recommended TC and LDL-C levels. However, only moderate doses of the different statins are used, with 76% of the patients on simvastatin taking 20 mg or less daily, 48% of atorvastatin users taking 10 mg daily, 55% of pravastatin users taking 20 mg daily, and 76% of rosuvastatin users taking 5 or 10 mg daily.

The subgroup of patients with type 1 diabetes was characterized by more renal disease but less history of CVD than the overall cohort. These patients were mostly treated with simvastatin or atorvastatin, and exhibited very similar LDL-C levels as the overall cohort but had higher mean HDL-C and lower TG levels, as expected.

Although around two thirds of the patients reach the overall European and Swedish treatment goal of LDL-C<2.5 mmol/L, many patients still have a high residual risk. The majority of patients had HDL-C above target levels and almost half of the population have elevated TG. Furthermore, in patients with a history of CVD, more than 70% do not reach LDL-C≤1.8 mmol/L. The treatment targets were thus not sufficiently achieved, particularly in the light of recently updated US and European treatment guidelines from year 2007 with a recommended goal for LDL-C of 2.5 mmol/L in patients with type 2 diabetes in general and 1.8 mmol/L in patients with a history of CVD [4], [5]. A slow improvement in overall risk factor control in Swedish patients with type 2 diabetes and coronary heart disease has been demonstrated, however, including an increased use of lipid lowering agents over time, with a corresponding improvement in blood lipid levels [17].

From 2003 and onwards generic simvastatin has been the first line choice of lipid lowering therapy. Other agents could be used when adverse effects appear, or if the individual treatment goals are not met. In this study there were only minor differences in patient characteristics between users of simvastatin, atorvastatin and rosuvastatin, apart from a slightly higher prevalence of a history of renal disease or CVD in the latter two. It is likely that a history of co-morbidities in the patients was the basis for the choice of statin in some cases, due to the presumed higher efficacy in atorvastatin and rosuvastatin. Still, the LDL-C levels are not lower than in patients taking simvastatin and the doses are low to moderate, suggesting that lipid lowering therapy is currently not consistent, and that a potential extra efficacy of atorvastatin or rosuvastatin has not been made use of [7], [8]. Furthermore, the results of the multivariate analysis taking clinical characteristics and LDL-C values before the treatment as well as doses of the statins into account, suggest similar LDL-C lowering effectiveness of these three agents. The weaker effects of pravastatin and fluvastatin in this study are in agreement with previous reports [7], [8], although our results must be interpreted with caution due to the small sample sizes and possible selection effects. Overall, these results from clinical practice verify a recent meta-analysis of published randomized clinical trials, showing that the different lipid lowering agents are equally efficacious at comparable doses [6].

A possible contributory cause for the results of this study could be the on-going discussion on the value of reaching certain treatment lipid goals vs. standardized treatment with statins in risk groups of patients, which could affect the prescribers. Major clinical trials such as the Heart Protection Study [1] and the Collaborative Atorvastatin Diabetes Study [2], underscored by the results of the recent meta-analysis by the Cholesterol Treatment Trialists' (CTT) Collaborators [3], have shown secondary preventive risk reduction after statin treatment also in patients without pronounced hypercholesterolaemia. In order to reduce CVD risk, however, the current US guidelines [4] promote statin use in patients with diabetes and overt CVD, or in patients without CVD who are older than 40 years and have one or more CVD risk factors. Alternatively, a reduction in LDL-C of 30–40% could be aimed at in patients not satisfactorily responding to a maximal dose of statin. The European guidelines [5] similarly promote LDL-C<2.5 mmol/L as the general treatment target in patients with type 2 diabetes or type 1 diabetes with nephropathy, but also give an opportunity for the clinician to offer statins in patients with LDL-C<2.6 mmol/L.

The NDR has currently an estimated coverage of more than 90% of all patients in hospital outpatient clinics and more than 70% of all patients in primary care. The patients included in this study are selected only based on completeness of the analysed data, suggesting that they are indeed representative. There might be minor errors in the clinical characteristics and risk factor values from clinics where these are reported manually, but more and more clinics transfer data automatically from computerized medical records systems. There were, however, some expected differences in mean levels and proportions of risk factors in the different treatment groups, suggesting possible selection effects. Therefore the results regarding blood lipid levels as well as the LDL-C lowering effects of the different treatments should be interpreted with some caution and should ideally be confirmed in prospective clinical trials.

All information on the lipid lowering agents is retrieved from Swedish Prescribed Drug Register, which contains complete information about drug utilization in the entire Swedish population [11]. We used strict criteria regarding the use of the lipid lowering treatments, with only patients without former purchases during a certain time period, followed by three purchases during a specified period of time. We used the blood lipid values reported after that period in our study, a technique that could cause some errors. We determined, however, this to be the best method to ensure the maximal number of patients in the study, since blood lipid values are not measured frequently in clinical practice, perhaps not more often than every second year in most patients, and they are not likely to be reported to NDR more than once every year.

In conclusion, this observational study shows that the LDL-C levels in patients taking simvastatin, atorvastatin or rosuvastatin are very similar as currently used, as well as their LDL-C lowering effects. In order to achieve better fulfilment of treatment goals, since the residual risk remains high in a large proportion of the patients, there is a potential to increase the doses of the lipid lowering treatments.

## Supporting Information

Table S1 Blood lipid values and history of CVD and renal disease of the patients with type 1 diabetes on lipid lowering treatment 2008.
(DOC)
