# Supplementary material for: Clinical Use and Effectiveness of Lipid Lowering Therapies in Diabetes Mellitus—An Observational Study from the Swedish National Diabetes Register
Source: PLoS One. 2011 Apr 29;6(4):e18744. doi: 10.1371/journal.pone.0018744 (PMC3084707; doi:10.1371/journal.pone.0018744)
Supplement: Table S1 — Blood lipid values and history of CVD and renal disease of the patients with type 1 diabetes on lipid lowering treatment 2008. (DOC) [file pone.0018744.s001.doc]

Table S1. Blood lipid values and history of CVD and renal disease of the patients with type 1 diabetes on lipid lowering treatment 2008

| Variable | | Simvastatin | Prava-statin | Fluva-statin | Atorva-statin | Rosuva-statin | Ezetimib | Fibrate | Statin + fibrate | Statin + ezetimib |
| --- | --- | --- | --- | --- | --- | --- | --- | --- | --- | --- |
| Number of patients | N | 2536 | 73 | 24 | 522 | 33 | 26 | 17 | 15 | 115 |
| TC | N | 2490 | 72 | 24 | 511 | 31 | 26 | 17 | 15 | 113 |
|  | Mean±SD | 4.5±0.8 | 4.8±0.8 | 4.9±1.1 | 4.6±0.8 | 4.9±1.2 | 5.3±0.9 | 4.8±0.7 | 4.9±1.2 | 4.6±1.2 |
| LDL-C | N | 2536 | 73 | 24 | 522 | 33 | 26 | 17 | 15 | 115 |
|  | Mean±SD | 2.4±0.7 | 2.7±0.6 | 2.8±0.8 | 2.4±0.7 | 2.7±1.1 | 3.1±0.8 | 2.7±0.6 | 2.7±1.0 | 2.4±1.0 |
| HDL-C | N | 2487 | 71 | 24 | 511 | 31 | 26 | 17 | 15 | 112 |
|  | Mean±SD | 1.6±0.5 | 1.6±0.5 | 1.4±0.5 | 1.6±0.5 | 1.6±0.5 | 1.7±0.5 | 1.5±0.5 | 1.2±0.4 | 1.5±0.5 |
| TG | N | 2483 | 72 | 24 | 511 | 31 | 26 | 17 | 15 | 113 |
|  | Mean±SD | 1.2±0.7 | 1.1±0.5 | 1.6±0.8 | 1.3±0.8 | 1.3±0.7 | 1.2±0.6 | 1.3±1.0 | 2.1±1.5 | 1.5±0.8 |
| CVD | N | 361 | 19 | 6 | 97 | 10 | 4 | 1 | 0 | 19 |
|  | % | 14.2 | 26.0 | 25.0 | 18.6 | 30.3 | 15.4 | 5.9 | 0.0 | 16.5 |
| Renal disease | N | 578 | 26 | 13 | 169 | 13 | 7 | 4 | 0 | 25 |
|  | % | 22.8 | 35.6 | 54.2 | 32.4 | 39.4 | 26.9 | 23.5 | 0.0 | 21.7 |

TC, total cholesterol; LDL-C, LDL cholesterol; HDL-C, HDL cholesterol; TG, triglycerides; CVD, history of cardiovascular disease; Renal disease, history of renal disease. SD, standard deviation.
